# Translation and Psychometric Testing of the Hägerbäumer Presenteeism Scale in English

**DOI:** 10.1007/s10926-024-10174-2

**Published:** 2024-03-11

**Authors:** Christoph Golz, G. Kilcher, M. Gerlach, M. Hägerbäumer, K. A. Peter, E. Blozik

**Affiliations:** 1 Department of Health Professions, Bern University of Applied Sciences, Bern, Switzerland; 2 Department of Health Services Research, SWICA Healthcare Organisation, Winterthur, Switzerland; 3 Department of Psychology, EURO-FH University of Applied Sciences, Hamburg, Germany; 4 Institute of Primary Care, University and University Hospital of Zurich, Zurich, Switzerland

**Keywords:** Presenteeism, Scale, Cognitive debriefing, Psychometrics

## Abstract

**Purpose:**

Interest in presenteeism has increased in research. Presenteeism is a behaviour of going to work despite illness. It has been predominantly measured using single items, which introduce limitations to validity. To overcome these limitations, Hägerbäumer developed a German multi-item presenteeism scale.

**Methods:**

The aim of the study was to provide an English translation and psychometric testing of the scale. This was conducted in two phases with native English-speaking employed adults. Phase 1 includes translation and cognitive debriefing, phase 2 testing construct validity and internal consistency reliability.

**Results:**

Cognitive debriefing with 10 employees revealed no problems with understanding or answering the translated items. In total, 487 employed adults participated in the study, of which data from 287 were included in the analysis. For structural validity, the goodness-of-fit indicators all reached their thresholds (TLI = 0.98, CFI = 0.99, RMSEA = 0.07, SRMR = 0.02). The scale does not show differences between sexes and age groups but between sectors (F_6,70.95_ = 5.53, p < 0.001). The internal consistency reliability was satisfactory with α = 0.89 (CI 95%, 0.87–0.91).

**Conclusion:**

The translated multidimensional scale for measuring presenteeism at the behavioural level demonstrated good psychometric properties in an initial validation. Further psychometric testing is required before using this scale in cross-national comparison in research and international companies.

**Supplementary Information:**

The online version contains supplementary material available at 10.1007/s10926-024-10174-2.

## Background

Interest in presenteeism both in applied research and in business contexts has increased, given its expected negative impact on both employee well-being and organizational productivity [1, 2]. For employees, presenteeism can negatively influence physical and mental health [3]. Several studies reported that the rising costs of lost productivity were predominantly caused by presenteeism [4–7]. The cost of presenteeism in the workplace accounted for 52% of the total cost of health-related production losses in Kigozi et al. [4]. However, in presenteeism research it is crucial to consider the underlying definition of presenteeism.

There are two dominant concepts of *presenteeism* [8, 9]: (1) The behaviour-oriented definition that defines presenteeism as the behaviour of going to work despite illness [3, 10]; and (2) the productivity-oriented definition that defines presenteeism as a productivity loss due to reduced performance of workers with untreated health problems [3, 11, 12]. While the definition of presenteeism as ‘a reduced performance at work, besides illness’ is predominantly used in studies in North America, the definition of presenteeism as a ‘behaviour of going to work despite illness’ has been used in European research [8, 9]. The current study focussed on the behaviour-oriented definition of presenteeism, as productivity loss is increasingly understood as a consequence rather than a definition of presenteeism [9].

Presenteeism is mostly assessed using employees’ self-reports [9]. Usually, single items are used that measure the absolute frequency of presenteeism behaviour in a certain reference period [9, 13], most commonly, the following single item from Aronsson et al. [14]: ‘Has it happened over the previous 12 months that you have gone to work despite feeling that you really should have taken sick leave because of your state of health?’ The response scale includes absolute frequency information (never, once, 2–5 times, more than 5 times), and the Likert scale is usually dichotomized. With this type of measurement, the base rate of disease incidence is included in the measured presenteeism frequency. People who were often ill significantly score higher on the frequency scale than people who were rarely ill. This leads to a confounding of behaviour and health status, so that the causes and effects of presenteeism cannot be examined separately from the behaviour being researched [13]. Furthermore, single items are prone to insufficient psychometric testing and are often outperformed by multi-item scales [15].

A first multi-item scale to measure presenteeism as a behaviour of going to work despite illness was developed by Hägerbäumer [13] in German. This scale addresses the confounding problem by measuring behavioural tendencies in relative terms by asking how often someone tends to work when ill. The scale initially demonstrated a high internal consistency reliability (α = 0.898), and an exploratory factor analysis revealed a one-dimensional construct with loadings above 0.73 and an overall 67% of explained variance [13]. It has been internationally recognized and recommended for translation: ‘Translating the [Hägerbäumer] scale or developing similar validated measures may help to establish a valid and reliable multi-item measure for presenteeism’ [9, p.349].

To contribute to the ongoing discussion in presenteeism research, complement the methodological inventory, and enable cross-cultural comparisons in research and international companies, the aim of this study was to translate the Hägerbäumer presenteeism scale into English and to test it psychometrically for construct validity and internal consistency reliability.

## Method

### Design

This study was conducted in two phases. First, the Hägerbäumer presenteeism scale was translated from German into English and tested using ‘cognitive debriefing’ in interviews. Second, the translated scale was psychometrically tested. For the reporting of the results, we adhered to the respective data extraction forms for internal consistency reliability, structural validity, hypothesis testing and cross-cultural validity (A, E–G) in the guidelines from the *Consensus-Based Standards of the Selection of Health Measurement Instruments* (COSMIN) [16].

### Phase 1: Cross-Cultural Validity

**Fig. 1:**
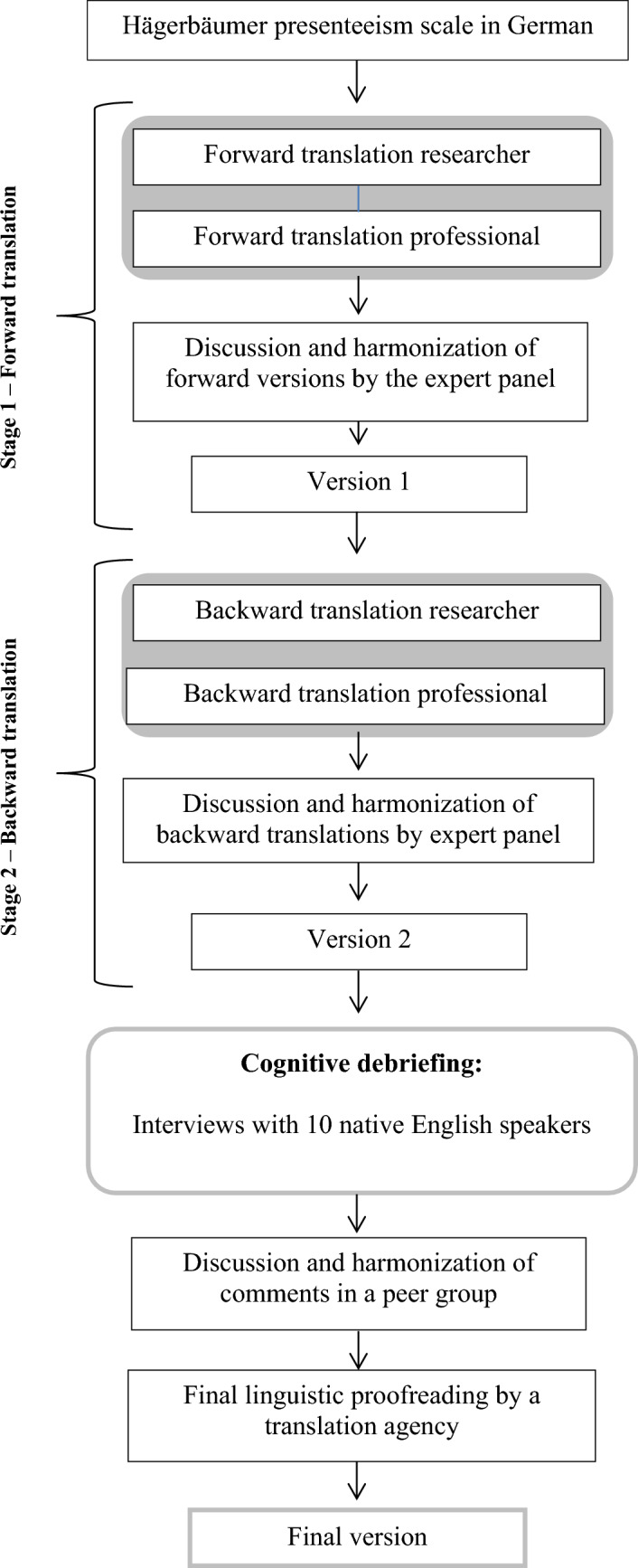
Methodological steps of translation and testing

To reach cross-cultural validity an adequate translation procedure is needed, followed by a statistical assessment of the original instrument’s psychometric properties, particularly the factor structure [16]. The Hägerbäumer presenteeism scale was translated according to the guidelines for scientific translation processes in *ISPOR Principles of Good Practice* [17]. Figure 1 summarizes the stages of the translation process. First, the items were independently forward-translated by a native English-speaking professional translator and a native English-speaking researcher. Then, the two versions were compared within an expert panel and a consensual final version 1 was created. The expert panel comprised the project team and the translators. Second, the translated items were independently back-translated into German by a native German-speaking translator and a native German-speaking researcher. Based on the back translation, the expert panel created the final version 2. Third, the final version 2 was tested for acceptability, understandability, and clarity using the method of cognitive debriefing [17]. Fourth, a final version was created and proofread by professional English translators.

#### Cognitive Debriefing

The final step in evaluating the items for acceptability, understandability, and clarity can be best achieved through interviews, as they account for individual subjective experiences [18]. To determine general comprehensibility, Collins' [19] cognitive interviewing was used in single interviews. Ideally, questions and answers should be clear and consistent within the target group to ensure the generalizability of the results [18]. Even seemingly reasonable answers might stem from a misunderstanding of the question [20]. Individuals might interpret questions and responses differently due to factors like language skills, jargon, education, and experience [18]. To answer a question, an individual must undergo four cognitive stages: (a) understanding the question and potential answer choices; (b) recalling relevant knowledge; (c) contemplating the intended response and whether to phrase it in a particular way; and (d) ultimately providing an answer [19]. For cognitive validation, the *verbal probing* technique was used. In this technique, answers are examined through one or more follow-up questions [21]. These additional enquiries were predetermined and included in an interview guide. In our interview guide, each question under evaluation was assigned one to three questions per stage, following Collins' [19] guidelines. See Table 1.

**Table 1:** Interview guide for cognitive debriefing according to Collins (2014)

Phase	Example questions	Problems
1. Understand question/answer	a. What do you understand by the term X? b. In your own words, what do you think this question asks?	The responder has difficulty understanding the question, a particular word or concept
2. Retrieve information	a. How easy or difficult did you find it to remember X? b. What time period did you think of when answering this question? c. When was the last time you did X?	The responder has difficulty retrieving information about the answer
3. Valuation of the answer	a. How did you elaborate your answer to this question? b. How accurate is your answer to this question? c. How did you feel when you answered this question?	The responder has difficulty formulating the answer
4. Answer	a. How easy or difficult was it for you to answer based on the available answer choices? b. Why did you choose this particular answer? c. Are you missing an answer category; if yes, which one?	The responder has difficulty giving the answer, or the possible answers do not apply

#### Recruitment and Study Sample

For cognitive debriefing, a minimum of ten individuals are needed [18, 19, 22]. Thus, native English-speaking employed adults from the network of the research group were personally contacted and asked to participate.

#### Data Collection

Data were collected in single interviews. If the interviewee had difficulties within one of the phases (1–4), field notes were taken by the interviewer. The interviews were recorded and transcribed using the F4-word processing program.

#### Data Analysis

The analysis of the cognitive debriefing interviews was conducted interpretatively using Microsoft Excel. The interpretative approach helps to gain ‘a more comprehensive understanding of a question’s performance [and] the question–response process’ [18, p. 37]. The identified difficulties were assigned to one of the cognitive stages.

### Phase 2: Psychometric Testing

The psychometric testing comprised structural validity, hypothesis testing, and internal consistency reliability checks.

#### Recruitment and Study Sample

To test structural validity using confirmatory factor analysis (CFA), 10 to 20 participants per item/question of a scale are recommended [23], which would lead to a minimum sample size of 60 to 120 for the six-item scale in focus. However, sample sizes below 150 are often found to be insufficient, and sample sizes above 300 are recommended [24]. Therefore, we aimed for a sample size above 300 participants. A combination of convenience and snowball sampling seeking native English-speaking employees between 18 and 65 years old from different sectors (e.g. assurance, construction, education) was used internationally to complete our online survey. Recruitment was carried out in two ways. A selection of 10 international companies based in Switzerland was identified via a website for expats in Switzerland (www.iamexpat.ch) and the respective Human Resource managers were invited to support the study. The inclusion criterion was that the company had its headquarters in an English-speaking country to ensure the highest possible proportion of native speakers. On the other hand, international research partners at universities in English-speaking countries were invited and were also asked to forward the survey to their existing contacts in companies.

#### Data Collection

Three companies agreed to forward the survey with the study information to their English-speaking employees. Emails with information about the study’s aim, inclusion criteria, data protection, and the survey link were sent to native English-speaking employed adults internationally. The participants were either reached by the supporting Human Resource managers or the researchers’ network by email and were asked to forward the invitation to their colleagues. Participation was voluntary.

##### Instrument

The survey included questions on individual characteristics (age, country, and profession) and the Hägerbäumer presenteeism scale that comprises six items based on a five-point Likert scale, with 1 = ‘Never in case of illness’, 2 = ‘Rarely in case of illness’, 3 = ‘Sometimes in case of illness’, 4 = ‘Often in case of illness’, and 5 = ‘Very often in case of illness’. In addition, it is possible to indicate that no illness occurred during the reference period (0 = ‘I was not ill’). The structural validity of the original scale was elaborated using exploratory factor analysis resulting in a one-factor solution, and confirmed using CFA with satisfactory comparative fit index (CFI) and Tucker–Lewis index (TLI) scores above 0.95 but a high Root Mean Square Error of Approximation (RMSEA) with 0.099 [13].

For interpretation of the Hägerbäumer presenteeism scale, a mean score is calculated per case. The scale score therefore has the same range as on the item levels between 1 = ‘Never in case of illness’ and 5 = ‘Very often in case of illness’. Cases with value 0 for at least one of the six items are excluded from the mean score calculation, since being not ill in the last 12 months would not classify as behavioural presenteeism based on the underlying definition.

#### Hypothesis Testing

Hypothesis testing is part of the construct validity [16]. We tested for known differences between different groups based on socio-demographic variables. Age and sex show heterogeneous effects but tend to show no relevant differences in terms of presenteeism [1]. This was also found by Hägerbäumer using the original instrument [13]. Furthermore, presenteeism differs across the sectors, whereas health professionals tend to have higher presenteeism scores [25]. Accordingly, the following three hypotheses were made:

##### H1

The means of the Hägerbäumer presenteeism scale do not differ significantly between sexes.

##### H2

The means of the Hägerbäumer presenteeism scale do not differ significantly between age groups.

##### H3

Health professionals have higher mean scores of the Hägerbäumer presenteeism scale compared to employees from other sectors.

##### Data Analysis

For the quantitative analysis, we used R 4.0.4 [26] and the packages ‘psych’ [27] and ‘lavaan’ [28]. Missing data were handled through list-wise deletion if at least one item was missing from the six items on the Hägerbäumer presenteeism scale [13].

Participants who responded that they had not been ill in the last 12 months were excluded from further analysis [13]. Descriptive statistics such as mean, median, standard deviation, minimum, maximum, skewness, and kurtosis were calculated. A correlation matrix was plotted for the six items and CFA was conducted to assess structural validity using robust maximum likelihood (RML) because the assumption of multivariate normal distribution of the items was not met and the data were defined as interval scaled [29]. Structural validity was estimated on the scale level by assigning the six to a one-factor solution to test the factor structure of the original instrument [13]. The values for standardized factor loadings were seen as satisfactory above 0.7 [30]. To evaluate the model fit, the following measures and cut-offs were used: a RMSEA below 0.05 was considered good, and values below 0.08 were deemed acceptable; a standardized root mean square residual (SRMR) below 0.08, and CFI and TLI scores above 0.95 were considered a satisfactory fit [23, 31, 32]. For hypothesis testing, the numeric variable age was categorized into four groups by using quartiles. Levene’s Test for homogeneity of variance was significant for the sector variable (H3). Therefore, analysis of variance with unequal variances with Games–Howell post hoc analyses was computed. For the other hypotheses (H1 and H2), analyses of variance with equal variances and the Tukey post hoc test for significant differences were applied. Because three hypotheses were tested on one data set, a Bonferroni-adjusted significance level of α = 0.017 (0.05/3) was calculated [33]. Cronbach’s alpha was used to test internal consistency reliability, with values greater than 0.7 considered satisfactory [34].

## Results

### Phase 1: Cognitive Debriefing

Overall, 10 native English-speaking employees participated in the single interviews. The interviews lasted 15 to 25 min. Half of the participants were female (50%) with an average 42 years (*SD* = 7.2). Participants worked in the healthcare (30%), education (30%), and research (40%) sectors. In the first round, all items were properly understood, and the participants answered according to the measure’s intention. One interviewee mentioned that she understood the word ‘illness’ as an acute abnormality from feeling healthy and that she would not consider her ‘chronic disease’, although it affects her sense of health. Referring to the underlying definition and considering the distinction between illness (a subjective experience) and disease (an objective professional classification), we did not consider a change of the term [35]. No other difficulties were identified; therefore, no adaptation and second round were needed.

### Phase 2: Psychometric Testing

In total, 487 employees responded to the online survey in January to April 2023. The mean age was 39 years (*SD* = 13). Most participants were from the United States (*n* = 324, 67%), followed by the United Kingdom (*n* = 53, 11%), Australia (*n* = 25, 5%), Switzerland (*n* = 22, 5%), Canada (*n* = 17, 3%), Ghana (*n* = 12, 2%), and Indonesia (*n* = 10, 2%). Most of the respondents were female (*n* = 269, 55%) and had a bachelor’s degree (*n* = 209, 43%), followed by participants with vocational education and training (*n* = 115, 24%). The sample is summarized in Table 2.

**Table 2:** Sample characteristics for the psychometric testing

Characteristics		Participants = 487		Participants included in the analysis = 287	
		Mean (*SD*)	*N* (%)	Mean (*SD*)	*N* (%)
Age		39.01 (13)		38.29 (13)	
Sex	Female		269 (55)		151 (53)
	Male		209 (43)		135 (47)
	Other		3 (< 1)		1 (< 1)
	Missing		6 (1)		
Education	No education		29 (6)		18 (6)
	Vocational education and training		115 (24)		37 (13)
	Bachelor’s		209 (43)		159 (55)
	Master’s		101 (21)		58 (20)
	PhD		30 (6)		15 (5)
	Missing		3 (< 1)		
Sector	Assurance		54 (11)		35 (12)
	Construction		37 (8)		29 (10)
	Education		78 (16)		55 (19)
	Finance		42 (9)		20 (7)
	Healthcare		140 (29)		103 (36)
	Information technology		21 (4)		12 (7)
	Retail		61 (12)		33 (21)
	Missing		54 (11)		
Country	United States of America		324 (67)		191(66)
	United Kingdom		53 (11)		43 (15)
	Australia		25 (5)		14 (5)
	Switzerland		22 (5)		13 (5)
	Canada		17 (3)		8 (3)
	Ghana		12 (2)		10 (3)
	Indonesia		10 (2)		8 (3)
	Missing		24 (5)		

Table 3 summarizes the descriptive results of the six items. Overall, 163 participants responded that they had not been ill in the past 12 months (value of 0) and were excluded from further analysis. Therefore, we proceeded with data from 324 participants. By handling missing data values with list-wise deletion, we computed the CFA with data from 287 participants, representing 89% of the participants, who reported that they had been ill in the past 12 months. The mean of the items ranged between 2.11 and 2.72. Skew and kurtosis were not found to be within the cut-offs, with <  ± 2 for skewness and <  ± 7 for kurtosis. For all items the whole scale ranging from 1 to 5 was used.

**Table 3:** Description of the items

No	Item	Mean	*SD*	Median	Min	Max	Skew	Kurtosis
1	I came to work despite illness	2.64	1.12	3	1	5	0.19	−0.69
2	I worked even though my doctor advised against it	2.11	1.20	2	1	5	0.78	−0.43
3	I worked in spite of showing more severe symptoms of illness (e.g. pain, chills, fever)	2.25	1.19	2	1	5	0.65	−0.46
4	I worked the full working day or the full shift despite illness	2.60	1.25	3	1	5	0.23	−1.04
5	Due to acute health problems, I took medication in order to be able to work	2.63	1.33	3	1	5	0.29	−1.07
6	Although I was ill, I dragged myself to work	2.72	1.24	3	1	5	0.17	−0.99

#### Structural Validity

The six items showed sufficient correlations ranging from 0.42 to 0.73. All goodness-of-fit indicators were above the defined thresholds. Table 4 summarizes the fit indicators for the one-factor solution.

**Table 4:** Goodness-of-fit indicators of the one-factor solution for the English Hägerbäumer (2017) presenteeism scale

Model	x^2^	df	x^2^/df	TLI	CFI	RMSEA	SRMR
One-factor	30.71***	9	3.41	0.98	0.99	0.07	0.02

***p < .001

The standardized factor loadings also reached the threshold of 0.7 to be acceptable (Table 5).

**Table 5:** Unstandardized and standardized loadings of the one-factor solution for the English Hägerbäumer (2017) presenteeism scale

Item	Unstandardized	Standardized
1	1.00	0.77
2	1.17	0.75
3	1.29	0.89
4	1.22	0.87
5	1.00	0.70
6	1.19	0.88

#### Hypothesis Testing

No significant difference of presenteeism was found between sexes (F_1,283_ = 0.025, p = 0.87), which leads to the acceptance of hypothesis H1.

No significant difference of presenteeism was found between age groups (F_3,283_ = 2.47, p = 0.061), which leads to the acceptance of hypothesis H2.

There was a significant difference of presenteeism between the sectors (F_6,70.95_ = 5.53, p < 0.001). The Games–Howell post hoc test showed a significant difference between employees in assurance and employees in healthcare sector, with a mean difference of 0.71 (95% CI, 0.16–1.25) which leads to the acceptance of hypothesis H3. Although presenteeism was slightly higher among construction workers, −0.09 (95% CI, −0.85 to 0.67) and employees in retail, −0.06 (95% CI, −0.72 to 0.60) compared to employees in healthcare sector, the mean differences were near zero with confidence intervals that span across zero.

The results of the hypotheses testing are summarized in supplementary file A.

#### Internal Consistency Reliability

The model showed high internal consistency reliability with α = 0.89 (CI 95%, 0.87–0.91). Cronbach’s alpha would not increase, if an item is dropped (0.85–0.87).

## Discussion

This study reports on the development and testing of an English version of the Hägerbäumer presenteeism scale [13]. The results show that the English version is construct- and cross-cultural-valid and has satisfactory internal consistency reliability. Based on a CFA and internal consistency reliability testing, the English translation compares well with the original German scale. However, in this study the RMSEA reached the threshold of < 0.8, which was not met for the German version from [13].

Until this study, the Hägerbäumer presenteeism scale had only been used among German-speaking participants. Therefore, only those results could be used for comparison. Regarding the descriptives of the single items, we found overall slightly higher mean values than Hägerbäumer [13]. The reason could be the similarity of the sample. The German scale has been psychometrically tested among health professionals. In our study, the proportion of employees from the healthcare sector made up 29% of the sample, who are prone to have higher presenteeism due to their working conditions [36]. This is underlined by the fact that we were able to prove in H3 that employees working in the healthcare sector report higher presenteeism scores. Furthermore, presenteeism is known to be associated with organizational factors, such as paid sick leave policy [10]. The policies for sick leave differ across countries, and this difference may have contributed to our findings, with more participants deciding to go to work despite their doctors’ advice. In our study, 67% of the participants stemmed from the United States, which is a country known to *not* have short-term paid sick days or longer-term paid sick leave as a requirement [37]. This contrasts with European countries that have explicit regulations in terms of compulsory sick pay insurance, for example [38].

### Implications for Research

To control for the country in which presenteeism is measured seems, thus, relevant, as lower presenteeism was found among employees with better compensation for illness-related absences [39, 40]. Furthermore, cultural differences between countries are known to influence presenteeism [10]. Our study sample is heterogeneous in terms of origin, but no information was collected on how conscientious respondents consider themselves to be or which occupational laws apply to them.

The three formulated hypotheses could be accepted, supporting the discriminative validity of the translated scale. However, hypothesis testing as part of the construct validity is a never-ending process [16]. Further analysis would be necessary to determine the extent to which the scale is able to differentiate between other known groups. Hägerbäumer [13] has generated various hypotheses, which should be validated using the English version of the scale.

Since the COVID-19 pandemic, a fundamental change has emerged that makes the understanding of work exclusively as a physical presence on-site obsolete and makes working remotely despite illness a new topic of presenteeism research [41, 42]. Some of the items in the Hägerbäumer presenteeism scale have in their wording the implication that work is done in person (only), and this might be an inadequate formulation for individuals working remotely. However, this limitation has been acknowledged and is under consideration [43]. Regarding the aforementioned limitation of the scale, a subsequent adaptation might incorporate an inclusive understanding of ‘work’, allowing the application of the scale in samples of individuals both working remotely or working while physically present [44].

### Implications for Practice

In recent times, the definition of presenteeism among researches seems to converge to the behaviour-oriented definition regardless of where the work is being done [9]. The productivity-oriented definition is increasingly viewed as a consequence of presenteeism [9]. The decision in projects or assessments in favour of one of the definitions is thus crucial for selecting appropriate measurement options. Ospina et al. [2] compared the psychometric properties of different scales that measure productivity loss or reduced performance. Therefore, these scales should be used in research that considers productivity loss as a consequence of presenteeism. For research with the underlying definition of presenteeism as a behaviour, the Hägerbäumer presenteeism scale is the first multi-item scale that offers an adequate basis for this purpose. The multi-item scale measures presenteeism in a behaviour-oriented and differentiated manner. The scale avoids mixing presenteeism behaviour with its antecedents (e.g. state of health) and consequences (e.g. health impairments, loss of productivity). This makes it a useful measurement tool in unconfounded research into the causes and effects of presenteeism.

### Strengths and Limitations

This study was based on the COSMIN [16] and *ISPOR Principles of Good Practice* [17] guidelines. Therefore, it used a standardized procedure for both translating a validated instrument and psychometrically testing the translation. Although the target sample size of 300 was not fully met, we came close to the intended number with 287 participants analysed, thereby providing a substantial basis for a sound statistical analysis. Furthermore, we recruited a heterogenic sample, which suggests that the scale can be used generically across work sectors.

Despite these strengths, the study has some limitations that should be considered. The convenience sampling method could have led to a sampling bias because we only included participants we could reach and who were willing to participate. Additionally, the broad sampling strategy resulted in a heterogeneous sample (e.g. culture, occupational law). The validity of the English scale may be, therefore, not given for homogeneous populations and it may differ in subgroups. In particular, since the cultural background and occupational law are known to play a crucial role in presenteeism, psychometric testing is needed for populations with different framework conditions. The German scale had already shown discriminative validity for known job demands and health-related consequences of presenteeism. It was also tested for convergence validity with the presenteeism single item by Aronsson et al. [14], with a moderate correlation of 0.50 [13]. Our study did not provide psychometric testing beyond determining its internal consistency reliability or testing construct validity with a CFA and hypothesis testing of three socio-demographic variables. Further testing should proceed with testing convergence or discriminative validity. Also needed is information about reliability aspects, such as test–retest reliability, as well as sensitivity to change. For cross-cultural studies using the Hägerbäumer presenteeism scale, psychometric comparison is needed beforehand, such as measurement invariance.

## Conclusion

We translated the German Hägerbäumer presenteeism scale into English and tested it psychometrically. We subsequently found that the scale is construct- and cross-cultural-valid and has satisfactory internal consistency reliability. Before it can be used to measure presenteeism among English-speaking employees, further psychometric testing is needed. The availability of an English version may facilitate the development of targeted interventions and contribute to the generation of new insights that will advance research on presenteeism.

## Supplementary Information

Below is the link to the electronic supplementary material.

Supplementary file1 (DOCX 38 KB)

## Data Availability

Data associated with this study are available upon reasonable request to the corresponding author.
