# Supplementary material for: Translation and Psychometric Testing of the Hägerbäumer Presenteeism Scale in English
Source: J Occup Rehabil. 2024 Mar 11;34(4):863–72. doi: 10.1007/s10926-024-10174-2 (PMC11550221; doi:10.1007/s10926-024-10174-2)
Supplement: Supplementary file 1 — Supplementary file1 (DOCX 38 KB) [file 10926_2024_10174_MOESM1_ESM.docx]

**Supplementary file A:** Results from the Post Hoc Analyses–

| Variable | Category | Difference | 95% CI | Adj. P |
| --- | --- | --- | --- | --- |
| Sector | Assurance vs. Construction | 0.80 | -0.05–1.65 | 0.08 |
|  | Assurance vs Education | 0.30 | -0.28–0.89 | 0.70 |
|  | Assurance vs Finance | -0.01 | -0.92– 0.90 | 1 |
|  | Assurance vs Healthcare | 0.71 | 0.16–1.25 | 0.004 |
|  | Assurance vs Information technology | -0.24 | -1.16–0.69 | 0.98 |
|  | Assurance vs Retail | 0.64 | -0.12–1.41 | 0.16 |
|  | Construction vs Education | -0.49 | -1.28–0.29 | 0.46 |
|  | Construction vs Finance | -0.81 | -1.84–0.23 | 0.22 |
|  | Construction vs Healthcare | -0.09 | -0.85–0.67 | 1.00 |
|  | Construction vs Information technology | -1.03 | -2.07–0.01 | 0.05 |
|  | Construction vs Retail | -0.15 | -1.07–0.77 | 1.00 |
|  | Education vs Finance | -0.31 | -1.16–0.54 | 0.90 |
|  | Education vs Healthcare | 0.40 | -0.01–0.81 | 0.06 |
|  | Education vs Information technology | -0.54 | -1.42–0.34 | 0.42 |
|  | Education vs Retail | 0.34 | -0.35–1.03 | 0.73 |
|  | Finance vs Healthcare | 0.71 | -0.11–1.54 | 0.12 |
|  | Finance vs Information technology | -0.23 | -1.31–0.85 | 0.99 |
|  | Finance vs Retail | 0.65 | -0.32–1.62 | 0.38 |
|  | Healthcare vs Information technology | -0.94 | -1.80– -0.08 | 0.03 |
|  | Healthcare vs Retail | -0.06 | -0.72–0.60 | 1.00 |
|  | Information technology vs Retail | 0.88 | -0.10–1.86 | 0.10 |
